# Skiing economy and kinematic during a field double poling roller skiing among novice and experienced cross-country skiers

**DOI:** 10.1038/s41598-024-57719-2

**Published:** 2024-03-25

**Authors:** Yang Zhu, Zhiqiang Wang, Ruoyang Li, Yanyan Li, Peng Bai, Weifeng Gao, Yaping Zhong

**Affiliations:** 1 Sports Big-data Research Center, Wuhan Sports University, Wuhan, China; 2 School of Competitive Sports, Shandong Sports University, Jinan, China; 3 School of Sports Economics and Management, Hubei University of Economics, Wuhan, China; 4 Department of Physical Education, Wuhan Sports University, Wuhan, China; 5 Department of Basic Science, Wuchang Shouyi University, Wuhan, China; 6 Hubei Sports and Health Innovation and Development Research Center, Wuhan, China

**Keywords:** Skiing economy, Kinematics, Double poling, Oxygen cost, Upper body strength, Physiology, Metabolism

## Abstract

To assess the skiing economy (SE) and kinematics during double poling (DP) roller skiing between two groups of skiers in a field setting. Five experienced and five novice male skiers performed a SE_DP_ test at 16 km∙h^−1^ on an outdoor athletics track. Gas exchange parameters were measured to determine SE_DP_. A two-dimensional video was filmed to measure the kinematics variables. Experienced skiers exhibited a 21% lower oxygen cost than novice skiers (p = 0.016) in DP, indicating a strong association between SE_DP_, cycle length and cycle rate (p < 0.001). Additionally, before the poling phase, experienced skiers manifested significantly greater maximum hip and knee extension angles than novice skiers (p < 0.001). During the poling phase, experienced skiers with a greater pole plant angle (p = 0.001), longer flexion time (p < 0.001) and higher flexion angular velocity in the elbow joint (p < 0.05) demonstrated better SE_DP_. There was an interaction effect of the one-repetition maximum bench press × group in SE_DP_ (b = − 0.656, SE = 0.097, t = − 6.78, p = 0.001). Therefore, experienced skiers with better SE_DP_ demonstrated more efficient cycles, potentially accomplished using dynamic full-body DP motion to ascertain effective propulsion. Combined upper body strength and ski-specific skill training may enhance SE_DP_ in novice skiers.

## Introduction

Skiing economy (SE), typically expressed as the steady-state oxygen uptake (VO_2_) required at a given sub-maximal skiing speed, may influence success in cross-country (XC) skiing performance. In the past two decades, scholars have shown increasing interest in this parameter^1–5^. In this regard, the primary sub-technique, double poling (DP), has been reported to be more cost-effective than other sub-techniques applied to flat sections and moderate slopes (with inclination at 4°–5°)^6^ and has become the predominant technique in classical XC skiing^7^. Furthermore, a previous study has demonstrated a high between-subject variability in DP SE (SE_DP_)^5^.

Previous studies in endurance sports have found that biomechanical factors (technique) make significant contributions to exercise economy among highly trained endurance subjects^8^. In XC skiing, a propulsive force is generated via symmetrical and synchronous pole movement, while skis glide continuously forward during DP^9^. Several studies on DP techniques have been limited to analyzing the kinematic characteristics of the Gross cycle^10–13^, joint and pole^5,14^, and center of mass (CoM)^5,15^. However, we only found two studies that have investigated the relationship between SE and the kinematics of DP. Zoppirolli et al.^5^ estimated that CoM’s more forward and less vertical displacement occurring at the beginning of the poling phase (PP) can explain approximately 73% of the variation of DP energetics. Therefore, utilizing the mechanical advantages of the CoM was suggested to improve poling effectiveness and decrease energy expenditure. Principal component analysis showed that a poorer SE was partially the result of superfluous movement, indicating that efficient skiers had a higher ability to reduce the residual movement during DP^15^.

Moreover, neuromuscular characteristics have been proven to improve the exercise economy by storing and releasing elastic energies in muscles and tendons, ultimately enhancing exercise efficiency^16^. DP techniques require developed upper-body strengths; therefore, the upper body might positively influence SE_DP_. However, studies have shown that heavy upper-body strength training had few^17–19^ or no effects^20,21^ on SE_DP_ in elite skiers. The differences among endurance sports might be due to the extreme technical demands of DP, which requires the skier to utilize both arms and legs sequentially before and during poling for optimization of propulsion^22^. As emphasized by these findings, “strong is not necessarily good” when the goal is to improve SE_DP_ in highly trained XC skiers.

Although this subject has been extensively researched, existing literature has some noteworthy limitations. First, while observations suggest that efficient propulsion technique may contribute to SE_DP_, the kinematic parameters of SE_DP_ remain unclear. Second, researchers generally conducted their studies by requesting skiers to simulate DP techniques on roller treadmills in a lab environment. A field setting increased the external test validity compared to lab-based tests; however, although limited SE research has been conducted outdoors during skiing on snow^1^ and on asphalt surfaces using roller skis^2^, to our knowledge, no such study is synchronously collecting whole-body kinematics data. Moreover, this kind of field test method faces difficulties such as unrepeatable test conditions and direct comparisons between studies. Finally, the question as to whether the general upper body strength is associated with SE_DP_ is yet to be investigated, especially for novice skiers.

Therefore, this study had two purposes: (1) to assess SE_DP_ and kinematics for non-homogeneous skiers on an outdoor standard 400-m athletics track and examine whether kinematics is related to SE_DP_ for the two groups, (2) to explore the influences of upper body strength level on SE_DP_ and determine whether these influences exhibit group-related differences.

## Results

Table 1 shows the descriptive features of the two groups of skiers. The experienced skiers (n = 5) were significantly older than the novice skiers (n = 5, p = 0.027) and had significantly more XC ski-specific training experience (p = 0.004). There was no significant difference (p = 0.987) in the data on body height and maximum VO_2_ (VO_2max_) between these two groups. The experienced male skiers had a greater body mass and were stronger at one-repetition maximum (1RM) bench press (BP) in terms of absolute and relative values and BP power (40% of 1RM BP), with highly significant differences (p < 0.001).

**Table 1:** Characteristics of the study participants.

	Skiers		t-value	p-value
	Experienced (n = 5)	Novice (n = 5)		
Age (years)	22.2 ± 2.6**	16.2 ± 0.5	5.108	0.001
Training (years)	9.2 ± 1.9**	2.6 ± 0.55	7.379	< 0.001
Body height (cm)	178.0 ± 3.9	177.8 ± 1.4	0.049	0.962
Body mass (kg)	70.0 ± 2.4	63.6 ± 1.4	2.268	0.053
VO_2max_ (mL·min^−1^·kg^−1^)	63.3 ± 6.5	62.5 ± 4.0	1.409	0.196
BP 1RM (kg)	99 ± 6**	53 ± 7	10.593	< 0.001
BP_rel_	1.42 ± 0.03**	0.83 ± 0.11	11.259	< 0.001
BP power (W)	788 ± 15**	327 ± 73	13.723	< 0.001

All outcomes are denoted as average values (standard deviation [SD]).

*VO*_*2max*_ maximum oxygen uptake, *BP 1RM* 1RM of bench press, *BP*_*rel*_ 1RM of bench press relative to body mass, *BP power* the maximal power output during a 40% load of 1RM in a bench press.

*p < 0.05, **p < 0.001. A p-value < 0.05 indicates statistical significance, while a p-value < 0.01 represents strong statistical significance.

Tables 2 and 3 present the physiological and cycle variables for the two groups. During the sub-maximal workload of 16 km·h^−1^, the VO_2_ of experienced skiers was significantly lower than that of the novice skiers by 21.1 ± 3.6% (p = 0.016, Cohen’s d = 0.77). We also observed significant differences between these two groups in heart rate (HR) (p < 0.001, Cohen’s d = 0.77), and blood lactate concentration [La-] (p = 0.011, Cohen’s d = 0.41). Moreover, the groups also manifested a large gap in the context of cycle variables at 16 km·h^−1^. The experienced skiers produced significantly greater cycle lengths (6.92 ± 0.46 vs. 5.07 ± 0.62 m, p = 0.001, Cohen’s d = 0.84) at a smaller cycle rate (39.59 ± 2.38 vs. 49.89 ± 4.13 cycles ·min^−1^, p = 0.002, Cohen’s d = 0.41). The DP cycle in this study comprised two phases, namely, the poling phase (PP) and recovery phase (RP). The recovery time for the experienced skiers was significantly longer by 26% of the cycle (1.13 ± 0.08 vs. 0.84 ± 0.09 s, p = 0.001, Cohen’s d = 0.86) than that of the novice skiers, whereas the relative poling time (26% vs. 29%, p = 0.067, Cohen’s d = 0.68) was shorter.

**Table 2:** Physiological response to double poling at sub-maximal speed (16 km·h^−1^) in the study groups.

	Skiers		t-value	p-value
	Experienced (n = 5)	Novice (n = 5)		
VO_2_ (mL·min^−1^·kg^−1^)	34.9 ± 1.3*	45.0 ± 5.8	− 3.817	< 0.05
RER	0.87 ± 0.03	0.89 ± 0.03	− 0.858	0.416
HR (beats·min^−1^)	139 ± 8**	165 ± 3	− 6.920	< 0.001
[La-] (mmol·L^−1^)	2.0 ± 0.2*	2.8 ± 0.4	− 3.697	0.011

All the outcomes are denoted as average values (standard deviation [SD]).

*VO*_*2*_ oxygen uptake, *RER* respiratory exchange ratio, *HR* heart rate, *[La-]* blood lactate concentration.

*p < 0.05, **p < 0.001. A p-value < 0.05 indicates statistical significance, while a p-value < 0.01 represents strong statistical significance.

**Table 3:** Cycle characteristics at sub-maximal speed (16 km·h^−1^) in the study groups during double poling.

	Skiers		t-value	p-value
	Experienced (n = 5)	Novice (n = 5)		
Cycle rate (cycles·min^−1^)	39.59 ± 2.38**	49.89 ± 4.13	− 4.827	0.002
Cycle lengths (m)	6.92 ± 0.46**	5.07 ± 0.62	5.363	0.001
Cycle time (s)	1.53 ± 0.09**	1.20 ± 0.10	5.097	0.001
Absolute poling time (s)	0.40 ± 0.02	0.35 ± 0.04	2.121	0.074
Relative poling time (%CT)	26 ± 1	29 ± 2	− 2.230	0.067
Absolute recovery time (s)	1.13 ± 0.08**	0.84 ± 0.09	5.292	0.001
Relative recovery time (%CT)	73 ± 2	70 ± 3	2.230	0.067

All the outcomes are denoted as average values (standard deviation [SD]).

*p < 0.05, **p < 0.001. A p-value < 0.05 indicates statistical significance, while a p-value < 0.01 represents strong statistical significance.

**Figure 1:**
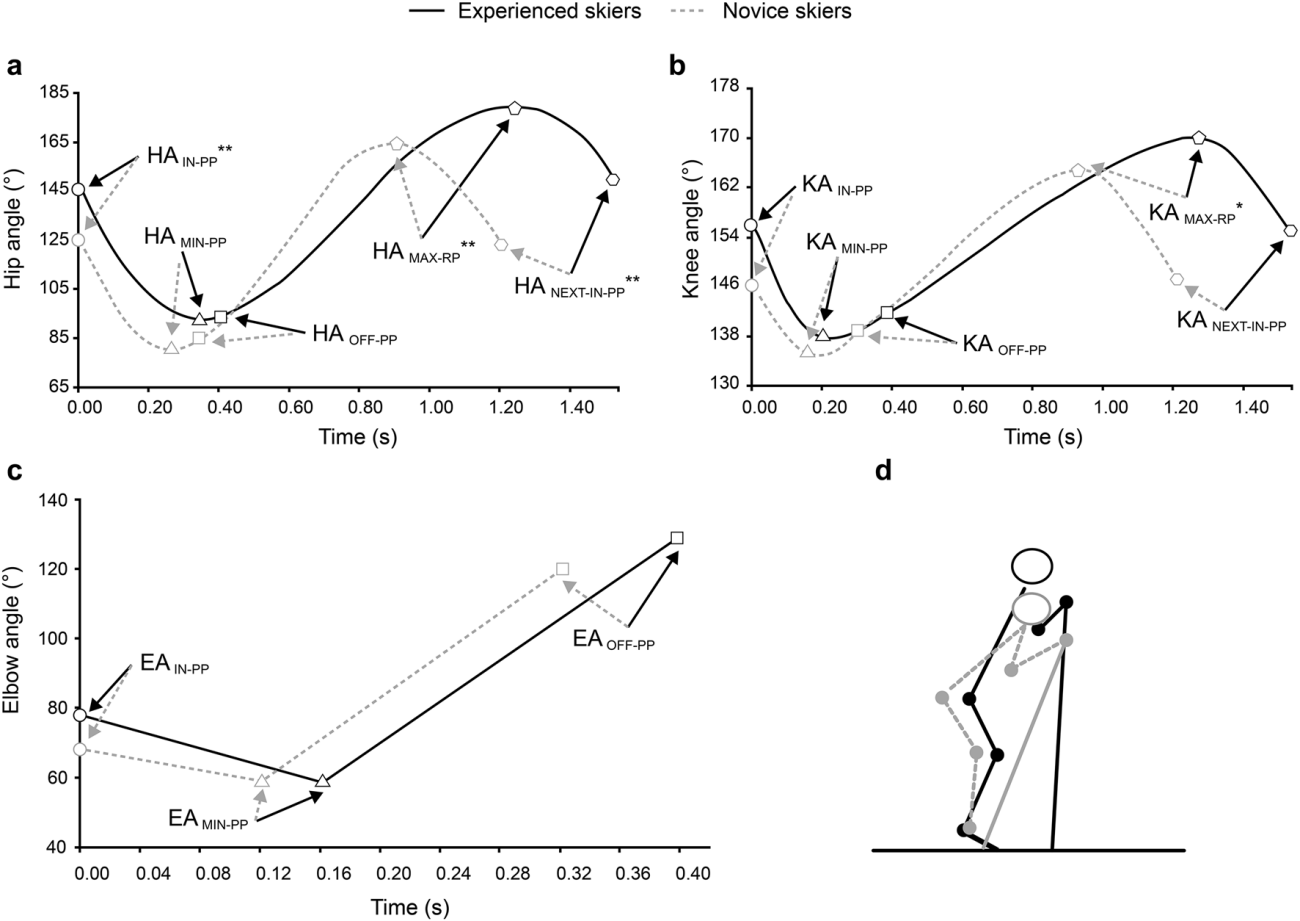
Measurements of the sub-maximal speed (16 km·h^−1^) test for experienced (n = 5) and novice (n = 5) skiers during double poling. Hip (**a**), knee (**b**), and elbow (**c**) angles at pole plant (IN-PP), angle minima value during the poling phase (MIN-PP), angles at the end of the poling phase (OFF-PP), and hip and knee angle maxima value during the recovery phase (MAX-RP). (**a**–**c**) Y-axes indicate mean angles (°) and X-axes represent time (s). (**d**) Mean body position of the two groups of skiers at the pole plant moment (bold lines denote the experienced skiers, and dotted lines denote the novice skiers).

Figure 1a–c and Table 4 present the pole and joint angle variables along with detailed statistics. All joint angle variables were gathered and calculated based on the joint angle curve within each cycle. We examined elbow, hip, and knee angles at particular movement events, including the start and end of pole ground contact, as well as the minimum and maximum values during the PP and RP. The 180° angle represented maximum elbow, hip, and knee extension. Pole angles (α°) were defined as the average pole inclination relative to the ground surface at the pole plant. During the PP, the phase between the minimum elbow angle and the pole plant was defined as elbow flexion, while the phase between the termination of pole ground contact and the minimum elbow angle was defined as elbow extension^23^. The onset of hip and knee flexion and extension was determined as the respective pole plant, the minimum value during the PP, and the maximum value during the RP. The ratio of the joint flexion–extension ranges to the joint flexion–extension times was set as the average angular joint speed. The data averages were calculated across two consecutive cycles on the right side.

**Table 4:** Inter-group differences in variable characteristics of the pole and joint angles and correlations with skiing economy during double poling at 16 km·h^−1^.

Parameter	Skiers		Skiing economy Spearman’s r (95% CI)
	Experienced (n = 5)	Novice (n = 5)	
Recovery phase			
T_H-EXT_ (s)	0.84 ± 0.11**	0.55 ± 0.11	− 0.933** (− 1.000 to − 0.556)
HA_MAX-RP_ (°)	180.76 ± 3.80**	165.39 ± 5.58	− 0.976** (− 1.000 to − 0.809)
T_K-EXT_ (s)	0.85 ± 0.18	0.61 ± 0.17	− 0.709* (− 1.000 to − 0.072)
KA_MAX-RP_ (°)	171.93 ± 3.03*	164.80 ± 3.81	− 0.782* (− 1.000 to − 0.259)
Poling phase			
α (°)	85.72 ± 2.08*	77.1 ± 2.97	− 0.939 (− 1.000 to − 0.644)
HA_IN-PP_ (°)	145.63 ± 9.54**	123.82 ± 10.01	− 0.806** (− 1.000 to − 0.427)
ROM_E-FLEX_ (°)	20.61 ± 2.96*	11.76 ± 2.69	− 0.685* (− 0.979 to − 0.176)
T_E-FLEX_ (s)	0.15 ± 0.01*	0.10 ± 0.01	− 0.688* (− 0.981 to − 0.021)
AV_E-FLEX_ [(°)·s^−1^]	131.71 ± 13.31*	108.28 ± 17.17	− 0.661* (− 0.987 to 0.154)
AV_E-EXT_ [(°)·s^−1^]	325.34 ± 26.75*	280.51 ± 12.07	− 0.176 (− 0.831 to 0.615)
Double poling cycle			
AV_H-EXT_ [(°)·s^−1^]	102.73 ± 10.57*	155.76 ± 29.23	0.903** (0.591 to 0.993)
AV_K-EXT_ [(°)·s^−1^]	35.75 ± 7.61*	40.50 ± 14.28	0.556 (− 0.712 to 0.669)

All outcomes are reported as means (standard deviation [SDs]). r, Spearman’s correlation coefficient, with 95% confidence intervals across 100 Bootstrap samples are presented.

*T*_*H/K-EXT*_ time for hip /knee extension during recovery phase, *HA/KA*_*MAX-RP*_ hip/knee angle maximum in the recovery phase, *HA*_*IN-PP*_ hip angle at pole plant, *α°* pole inclination relative to the ground surface, *ROM*_*E-FLEX*_ elbow flexion range of motion in the poling phase, *T*_*E/H-FLEX*_ time for elbow/hip flexion in poling phase, *AV*_*E-FLEX/EXT*_ angular velocity of average elbow flexion/extension in the poling phase, *AV*_*H/K-EXT*_ angular velocity of hip/knee extension during double poling cycle.

*p < 0.05, **p < 0.001. A p-value < 0.05 indicates statistical significance, while a p-value < 0.01 represents strong statistical significance.

Inconsistent angular patterns were observed among the subgroups. During the RP, the maximum hip and knee extension joint angles were significantly larger (p = 0.007 and 0.012, Cohen’s d = 0.86 and 0.72, respectively) in experienced skiers than in novice skiers. At the moment of pole plant, the hip joint angles and α° of the experienced skiers were significantly greater (p = 0.008 and 0.001, Cohen’s d = 0.74 and 0.86, respectively) than those of the novice skiers (Table 4). The elbow joint angle was greater in the experienced group than in the novice group, while the minimum elbow joint angle was smaller than that of the novice group during the PP (Fig. 1c). This resulted in a significantly longer flexion time (p < 0.001, Cohen’s d = 0.93), greater flexion range (p = 0.001, Cohen’s d = 0.83), and higher flexion angular velocity (p = 0.042, Cohen’s d = 0.60) at the elbow angle.

When the pole and joint angles were evaluated during the cycle, clear relationships were observed between SE_DP_, hip, knee, and elbow joint angles and α°. The strength of these relationships is described in detail in Table 4. The maximum hip extension angle showed a significant relationship with α° (r = 0.939, p < 0.001), flexion range (r = 0.855, p < 0.001), flexion time (r = 0.890, p < 0.05), and angular velocity (r = 0.648, p < 0.05) in the elbow. There was a positive correlation between the absolute poling time and the absolute recovery time (r = 0.658, p < 0.05), α° (r = 0.737, p < 0.05), the maximum hip extension angle (r = 0.659, p < 0.05), and the flexion time in the elbow (r = 0.698, p < 0.05).

Table 5 displays the correlation among cycle variables, upper body strength, and SE_DP_ at 16 km·h^−1^ in experienced skiers (n = 5), novice skiers (n = 5), and the entire sample (n = 10). However, upper body strength was closely associated with SE_DP_ only in the entire sample (p < 0.001) and in novice skiers (p < 0.05).

**Table 5:** Correlations among gait cycle variables, upper body strength, and skiing economy at sub-maximal speed (16 km·h^−1^) during double poling in experienced skiers (n = 5), novice skiers (n = 5), and the whole sample (n = 10).

	Novice	Experienced	Whole sample
Poling time (s)	− 0.873 (− 1.000 to 1.000)	− 0.564 (− 1.000 to 0.983)	− 0.558 (− 0.895 to 0.163)
Recovery time (s)	− 0.931** (− 1.000 to − 0.792)	− 0.900* (− 1.000 to − 0.250)	− 0.988** (− 1.000 to − 0.887)
Cycle rate (cycle·min^−1^)	0.983** (− 1.000 to 1.000)	0.900* (− 0.370 to 1.000)	0.964** (0.747 to 1.000)
Cycle length (m)	− 0.950* (− 1.000 to 1.000)	− 0.900* (− 1.000 to − 0.190)	− 0.964** (− 1.000 to − 0.768)
BP 1RM (kg)	− 0.900* (− 1.000 to − 0.250)	− 0.700 (− 1.000 to 0.757)	− 0.952** (− 1.000 to − 0.691)
BP power (40% 1RM)	− 0.900* (− 1.000 to − 0.191)	− 0.500 (− 1.000 to 0.929)	− 0.927** (− 1.000 to − 0.506)

Data are Spearman’s ρ correlation coefficients, with 95% confidence intervals across 100 Bootstrap samples are presented.

*BP 1RM* a one-repetition maximal value for bench press, *BP power* the maximal power output during the 40% of 1RM in a bench press.

*p < 0.05, **p < 0.001. A p-value < 0.05 indicates statistical significance, while a p-value < 0.01 represents strong statistical significance.

The results from the linear regression analysis underscore the significant impact of both the group and 1RM BP factors on SE. The model demonstrated an exceptional fit, with an adjusted R^2^ of 0.980 (F = 147.97; p < 0.001), suggesting that 98% of the variability in VO_2_ can be explained by the predictors included in the model. The main effects and interaction were evaluated at a significance level of 0.05. The group variable, representing the difference in XC ski-specific training experience between groups, was significantly associated with VO_2_ (b = 39.548, SE = 8.067, t = 4.9, p = 0.003). This result indicates that experienced skiers have a higher SE than novice skiers. Upper body strength level, measured by 1RM BP, was not significantly associated with VO_2_ (b = − 0.116, SE = 0.074, t = − 1.58, p = 0.165), suggesting that without considering XC skiing-specific experience, upper body strength alone does not predict SE_DP_.

The interaction between the 1RM BP and group factors was significant (b = − 0.656, SE = 0.097, t = − 6.78, p = 0.001), indicating a differential effect of upper body strength on VO_2_ across XC ski-specific experience levels. Specifically, the negative coefficient of the interaction term implies that for experienced skiers, increases in general upper body strength are associated with a smaller increase in SE_DP_ than that for novice skiers. The 95% confidence intervals for this interaction term (− 0.893 to − 0.419) did not contain zero, further supporting the significance of this effect. Additionally, the constant term (b0 = 46.494, SE = 7.331, t = 6.34, p = 0.001) indicated the baseline level of VO_2_ when the 1RM BP and group factors were at their reference levels.

Post hoc power analysis using G*Power (v.3.1) obtained an effect size of 2.40 at a power of 0.91 at an α of 0.05 for a group of 10 participants.

## Discussion

This study is the first to explore the relationship between O_2_ cost and kinematic parameters on outdoor athletics tracks in two groups of skiers with varying performance levels. As a result of the kinematic parameters measured here, we observed a higher SE_DP_ level among experienced skiers than their novice counterparts.

Previous studies have reported that O_2_ cost is influenced by the performance level in XC skiing^3,4,24^. In our study, experienced skiers exhibited 21% less O_2_ cost at sub-maximal workloads compared to novice skiers during DP. Our results are consistent with previous findings showing that national skiers expend up to 30% less O_2_ cost compared to regional skiers when skiing at the velocity of 14 km·h^−1^ during DP^5^. Another study also reported that elite skiers exhibit only 4–15% lower O_2_-cost compared to their novice counterparts during sub-maximal diagonal stride skiing and skating^4^. These results suggest that the differences in SE_DP_ tend to be greater in a heterogeneous sample of skiers when the upper body movement is more involved, thereby highlighting the significance of movement efficiency during DP. Although older age or longer ski-specific training experience likely explains the SE_DP_ differences between the groups, the kinematics exhibited a significant difference between the two groups in our study.

Our study revealed a significant correlation between SE_DP_, greater cycle length, and smaller cycle rate at a given speed. These cycle characteristics were also observed in top-performing skiers who used DP at sub-maximal speeds^25,26^. From a mechanical standpoint, it is estimated that the upper body of a male elite XC skier occupies 68% of his total body mass^27^, with the lower cycle rates during DP potentially decreasing internal work and reducing energy costs by approximately 30% per cycle^25^. From a physiologic point of view, longer cycle lengths indicate an increase in swing and muscle recovery times, which may facilitate blood perfusion of the exercising muscle and clearance of blood lactic acid^11,28^, thereby decreasing anaerobic energy consumption.

Notably, we found minor between-group differences (12%) in absolute poling time; moreover, experienced skiers can produce longer swing times than that of novice skiers, similar to findings from previous studies. That study pointed out that the absolute poling time increased (17%) with a decrease in poling frequencies^23^ at 18 km·h^−1^. This may be an effective discriminator during DP at low poling frequencies compared to high frequencies, based on a more extended period of force application and better technique-specific propulsion^29^. Previous studies have demonstrated that longer pole ground contact time can be associated with a larger plant angle with respect to the ground, up to 90°, and a higher position for the COM^5^ in elite skiers. This finding is partly confirmed by our results showing a greater maximum hip and knee extension angle and a greater α° in experienced skiers. Furthermore, both were positively correlated with the absolute poling time.

From a technical strategies standpoint, experienced skiers adopted a highly dynamic full-body DP motion (Fig. 1d) based on the results of the above analyses, with the use of lower limb flexion and extension motions transferring the potential energy of the increased body mass to the pole^30^. Potential energy fluctuations were not measured in this study, although this mechanism may be relevant here. Conversely, novice skiers exhibited an upper body DP motion (Fig. 1d), mainly relying on shoulder and elbow flexion and extension movement for propulsion. Previous research investigated how lower-body movement influenced DP performance through a comparison of the upper body (locking the knee and ankle joints) with whole-body DP motion performed by the same XC skiers. The results showed that active leg movements could noticeably increase the efficiency of DP skiing due to appropriate body repositioning within a motion cycle. However, upper body DP motion induces a greater relative load on the upper limb muscles, which may limit the perfusion of the working muscles^28^ and elevate [La-]^11^. In this study, novice skiers had higher [La-], thus supporting this explanation.

Continuing during the PP, it is interesting to note that elbow joint flexion ranges were 50% larger and extension angular speed was 14% higher in experienced skiers than those in novice groups, both having significant correlations with SE_DP_. We speculated that this active pattern in the elbow joint, particularly in the lower cycle, may be attributed to the patterns of the stretch–shortening cycle (SSC) of the elbow joint and the need to generate higher forces to gain a longer recovery time. Distinct SSC in the elbow joints during DP has been demonstrated previously^31–34^ and is considered an essential characteristic of DP performance^25^. Existing evidence suggests that the SSC can improve the work economy in endurance sports. During the flexion phase, the high stretching velocities of the muscle–tendon complex reflect higher muscle stiffness and storage of elastic energy^35^. Consequently, during the concentric phase, the lower motor unit activity in the triceps muscle can increase mechanical efficiency^31^, decrease energy expenditure in the muscles^35,36^, and optimize human locomotion^32^. To the best of our knowledge, the present study is a pioneering study that demonstrates a significant correlation between elbow flexion and SE_DP_; however, further research will be needed to understand the function of elbow SSC in SE_DP_.

Further analysis demonstrated that the maximum hip and knee extension joints with a greater vertical α° prior to and at the beginning of the PP, greater flexion time and amplitudes, and higher extension angular velocities at the elbow during the PP were related to SE_DP_ and also correlated with each other. Such findings contribute to a more comprehensive understanding of the dynamic full-body DP motion between the pole and upper and lower limbs and can help ascertain the effective propulsion determinants of SE_DP_.

Additionally, larger individual variations were observed in SE_DP_ in novice skiers than in experienced skiers. This result was inconsistent with that of a longitudinal analysis^37^ proposing that SE was not a discriminating factor among young XC skiers. These contrasting results may be related to differences in the experience level of study participants. High-level adolescent XC skiers participated in the study by Zoppirolli et al*.*^37^, whereas the participants in the present study were transferring athletes with experience in middle- and long-distance running. As these participants were already well-developed in endurance capacities after 3 years of general endurance training, SE_DP_ could distinguish sport-specific performance within this homogeneous group.

We observed a statistically significant interaction between the group and 1RM BP on SE_DP_. This finding may be due to the higher demands of technical capabilities by DP than a general upper body strength level in well-trained XC skiers, with the rationale that general upper body strength gained does not translate directly into technique-specific propulsion^20,21^. However, it is worth noting that upper body strength is an important influencing factor on SE_DP_ in novice skiers. A recent longitudinal study following Chinese talent transfer XC skiers with backgrounds in various summer endurance sports confirmed that the development of DP efficiency was coupled with improved strength, power, and cycle lengths over a 6-month training period^38^. Therefore, the practical recommendation is to individualize the focus when supplementing strength training to improve SE_DP_. Combining upper body strength and ski-specific skill training programs to lengthen the cycle may help improve SE_DP_ in novice skiers.

This study had a few limitations. First, the relatively small and heterogeneous sample may restrict the universality of the results for other XC skiers. Second, although upper body strength confounders were considered in this study, there was a lack of information on biomechanical factors, including muscle activation patterns, pole forces, tendon stiffness, and tendon function; thus, we could not completely rule out the possibility of unmeasured confounding. Moreover, the cross-sectional study failed to clarify the causal association, necessitating more prospective cohort research and intervention trials that can establish a causal relationship between SE_DP_ and biomechanical factors and neuromuscular characteristics.

In conclusion, this is the first study to characterize the kinematic factors associated with SE_DP_ in relatively heterogeneous groups of male XC skiers on an outdoor flat ring track. The improved SE of experienced skiers may be attributed to a dynamic full-body DP motion, helping to ascertain the effective propulsion determinants of SE_DP._ However, we observed a statistically significant interaction effect between group and 1RM BP on SE_DP_. Understanding these factors may assist coaches in developing techniques and interventions for strength training to optimize movement patterns and reduce O_2_ cost during DP. In addition, the field-based SE_DP_ test has exhibited promising potential as a monitoring tool for physiological and technical training programs.

## Methods

### Ethical considerations

The present study was approved by the Ethics Committee of Wuhan Sports University (approval number 2022042) and was conducted according to the tenets of the Declaration of Helsinki. Before data collection, written informed consent was obtained from each skier and parents of participants below 18 years of age.

### Study participants

Ten XC skiers from the Jilin Changchun Winter Sports School were recruited for this study between June 2022 and July 2022. For inclusion in the study, all male skiers were required to have a VO_2max_ > 60 mL∙kg^−1^∙min^−1^. The study participants were allotted into two groups, i.e., the experienced (n = 5) and novice (n = 5) groups, according to their XC ski-specific training experience. Experienced skiers had training experience of ≥ 6 years and had competed at the national level. The novice skiers were young Chinese transfer athletes with middle- and long-distance running backgrounds, with a training history of ≥ 3 years. Novice skiers were required to have ≥ 1 year of experience in XC skiing and to have competed at the regional level. Each participant was familiar with DP techniques on the outdoor athletic track and was free of any injury or illnesses for at least 4 weeks before testing.

### Study design

All participants were evaluated twice, at an interval of at least 2 days. During the initial evaluation, participants were tested for upper body strength and treadmill incremental test under laboratory settings. On subsequent evaluation, SE_DP_ field testing and two-dimensional (2D) kinematic characteristics analysis were performed on an outdoor standard 400-m athletics rubber track. All tests were undertaken during the summer season.

### Upper body strength test

Tests of upper body strength in the laboratory included 1RM BP and BP power. BP is commonly used to assess strengths and analyze the functional movement patterns during DP, as recruited muscles in exercise have been shown to be active in DP^25^.

The 1RM BP protocols were performed as follows: (a) warm-up using a weight of 30–50% weight of the estimated 1RM BP for 10 repetitions; (b) 1 min rest and repeated five, three, and two times at an estimated 60%, 70%, and 80% of 1RM, respectively; (c) after 2–3 min of rest, single repetitions were performed at an estimated 85%, 90%, 95%, and 100% of 1RM. The participants were given up to three opportunities to achieve their 1RM BP, with loads increasing by 0.5–5 kg and 5-min rest intervals to ensure adequate recovery. Two independent researchers determined 1RM BP based on the heaviest accepted trial and evaluated the results. Following a recovery for at least 20 min, participants began a self-administered warm-up lasting for 10 min prior to the BP power measurement. The participants performed explosively for three consecutive repetitions at 40% of their 1RM BP^39^. BP power was measured using GYM (GymAware Power Tool, Kinetic Performance Technologies, Canberra, Australia) and was characterized as the maximal value during the concentric phase at 40% of 1RM BP.

### Treadmill incremental tests

A test of the incremental exercise was performed till exhaustion on a calibrated treadmill (RL 1700 treadmill; Rodby Innovation AB, Sweden) to determine the VO_2_max. According to protocols in a previous report, a conventional monitoring method for XC skiers was used^40^. The participants warmed up for 10 min before the test with their customized running speeds on the treadmill. Subsequently, the test was conducted at a fixed inclination of 10.5%, starting with the customized initial running speed and a step increase of 1 km·h^−1^ per minute, and the duration was 6–8 min. Maximal aerobic capacity was considered to be achieved when two of the following three conditions were satisfied: (a) a VO_2_ plateau as the exercise intensity increased, (b) a respiratory exchange ratio over 1.1, and (c) a peak lactate value beyond 8 mmol·L^−1^. Oxygen uptake measurements were taken continuously, and the three highest 10-s successive results were averaged to obtain VO_2_max. The analyzer was calibrated before each test using an ambient atmosphere with known O_2_ (16.00 ± 0.04%) and CO_2_ (5.00 ± 0.10%) contents, whereas the speed of the treadmill was calibrated using an inbuilt speedometer mounted on the treadmill platform at the start of the testing day.

### SE_DP_ field test

The SE_DP_ field test sessions were conducted on an outdoor standard 400-m athletics rubber track over 2 consecutive days from 08:00–12:00 under similar weather conditions (temperature: 27 ± 2 °C; humidity: 68 ± 4%). During the SE_DP_ field testing session, all skiers used the same model of roller skis (Nord, Ski Skett, Sandrigo, Italy) and carbon fiber poles (One Way, Diamond Storm, Helsinki, Finland), although pole lengths were adjusted according to their individual preferences.

Following a 15-min warm-up period at an intensity of 60–70% of peak HR DP roller skiing, participants were instructed to complete three-lap DP roller skiing trials at 16 km·h^−1^, a velocity chosen based on a pilot test. Additionally, this velocity provided novice skiers with a comfortable and sustainable pace while providing an engaging exercise intensity in both groups. Before the data collection, each subject performed two practice DP roller skiing trials at 16 km·h^−1^ on the outdoor athletic track to familiarize themselves with the desired speed. The participants were paced by a technician who carried a calibrated electronic speedometer on a bicycle and rode in front of the participants^1,41^. The speed was measured with a stopwatch according to the time used to complete each course lap. The speed for each loop was different, with an average value of about 0.4 km·h^−1^ deviating from the ideal speed.

The cardiorespiratory variables VO_2_, respiratory exchange rate (RER), HR, and ventilation volume (VE) were continuously monitored during the SE_DP_ field test with the aid of an easy-to-carry metabolic system (Cosmed K4b^2^, Rome, Italy). We allotted three-lap DP roller skiing lasting approximately 4.5 min, allowing participants’ VO_2_ to reach a steady state, defined as the plateau in O_2_ uptake that is reached following a few min of exercise. VO_2_ may be altered by < 10% during the final 1 min, and the RER may drop below 1.0 at a given speed. The average VO_2_ (mL·kg^−1^·min^−1^) values gathered at the last minute were considered. Blood was collected from a finger prick immediately following the completion of the test, and a portable Lactate Pro LT-1710t (ArkRay Inc, Kyoto, Japan) was then used to measure [La-]. Participants were allowed to use personal poles but were instructed to use the same pair of roller skis during the SE_DP_ field test sessions to reduce differentiation in roller resistance.

### 2D kinematics

2D kinematic data was simultaneously recorded when VO_2_ reached a steady state on the 3rd lap using four video cameras (Sony HDR-PJ810E, Sony Corp., Tokyo, Japan) with a sampling rate of 60 Hz and a shutter speed of 1/500 s. The cameras recorded the skier in the sagittal plane at high resolution (1920 × 1080 progressive scan)^42^. The optical axes of the four cameras were vertically fixed at 1.4 m above the ground and located parallel to the direction of roller skiing at a distance of 14 m from the middle of the track. The cameras were positioned at 55, 60, 65, and 70 m of the 100 m runway, respectively. Each camera had a field of view of 10 m, resulting in a total camera field of 25 m, which is necessary to capture at least two full cycles in reference to cycle lengths previously reported in the literature^13,43^. Before the start of the SE_DP_ field test, four filming sections were calibrated carefully. Two cones with a 200 cm-long calibration stick on top were placed alongside the middle of the track in each 10-m filming section. Thereafter, four short calibration videos were recorded. Details of the calibration procedures have been described in a previous study^44^. The horizontal axis was aligned with the direction of roller skiing, while the vertical axis was set perpendicular to the track. The camera positions and filming conditions were maintained after calibration. The video recording showed that none of the skiers deviated from their track.

To ascertain the kinematics variables of the sagittal plane on the right side, 2D video footage was processed and converted to 60 Hz using the SIMI Motion software (SIMI Motion Systems, SIMI, Germany). For each camera view, 12 points were digitized^43^, and cycle characteristics along with the pole and joint kinematics were obtained manually by an XCS expert through frame-by-frame analysis. The kinematics data were filtered with a second-order Butterworth filter (cut-off frequency 10 Hz).

For this study, one DP cycle was separated into the PP and RP. The period of right pole ground contact represented the absolute poling time. The period of poles off the ground represented the absolute recovery time. CT refers to the time interval between two subsequent right pole ground contacts. The period of arm swing represented the absolute recovery time. The relative poling/recovery time was the absolute poling/recovery time ratio to CT (%CT). The cycle index included CT (s), cycle rate (cycles time·min^−1^), cycle length (CT·velocity), relative (%CT), and absolute (s) poling and recovery time.

The angle of joints and poles were assessed in the sagittal plane and were calculated in two dimensions. We examined elbow, hip, and knee angles at particular movement events, such as pole plant and pole off, as well as the minimum and maximum values during the PP and RP. The pole angles (α°) were defined as the average pole inclination relative to the ground surface at the pole plant. The angle of 180° represented the maximum extension angle of the elbow, hip, and knee joints, and the thighs were aligned with the trunk. During the PP, the phase between the minimum elbow angle and the pole plant was defined as elbow flexion, while that between the termination of pole ground contact and the minimum elbow angle as elbow extension^24^. The beginning of hip and knee flexion and extension was determined as the respective pole plant, the minimum value amidst the PP, and the maximum value amidst the RP. The ratio of the joint flexion–extension ranges to the joint flexion–extension times was set as the average angular speed of the joints. The data averages were calculated across two consecutive cycles on the right body side.

### Statistical analysis

All the outcomes are denoted as an average value (standard deviation [SD]). The Shapiro–Wilk test was conducted to evaluate data normality. Training experience was considered as a grouping variable, and the differences between the two groups for all variables were compared through an independent *t*-test. It was reported that the effect size was Cohen’s d (a value of 0 < d < 0.2 corresponded to a very small effect; 0.2 < d < 0.5, small; 0.5 < d < 0.8, medium; d > 0.8, large)^45^. Spearman’s correlation was extracted with 95% confidence intervals and corresponding p-values to investigate the relationship between SE_DP_ and 2D kinematic variables. A p-value < 0.05 represents statistical significance, while a p-value < 0.01 represents strong statistical significance. The degree of correlation was evaluated based on six scales: extremely large (0.9–1.0), very large (0.7–0.9), large (0.5–0.7), moderate (0.3–0.5), small (0.1–0.3), and trivial (0.0–0.1)^46^.

A linear regression model with an interaction term was created to identify between-group differences in the effect of 1RM BP on SE_DP_:

$$ {\text{y}} = {\text{b}}0 + {\text{b1x1}} + {\text{b2x2}} + {\text{b3x1x2}}, $$

where y represents VO_2_; x1 and x2 are the independent variables representing the BP 1RM and group factors, respectively; b0 is a constant coefficient; b1 and b2 are the linear coefficients; and b3 is the interaction factor coefficient. The interaction effect size (based on SMD) and its 95% confidence interval were adopted to ascertain the type of interaction. The statistical analysis was mostly performed using IBM SPSS Statistics 24.0 (IBM Corp., Armonk, NY), and the linear regression analysis was conducted using Stata 12.1 (Stata Corporation, College Station, TX).

Post-hoc power analysis was conducted utilizing G*Power 3.1 software44 to determine if satisfactory effects could be obtained using the specific sample size.

### Supplementary Information

Supplementary Information.

## Data Availability

This published article includes all the data generated or analyzed during this study (and its Supplementary Information files).
