# Author Correction: Skiing economy and kinematic during a field double poling roller skiing among novice and experienced cross-country skiers

**DOI:** 10.1038/s41598-024-59541-2

**Published:** 2024-04-15

**Authors:** Yang Zhu, Zhiqiang Wang, Ruoyang Li, Yanyan Li, Peng Bai, Weifeng Gao, Yaping Zhong

**Affiliations:** 1 Sports Big-Data Research Center, Wuhan Sports University, Wuhan, China; 2 School of Competitive Sports, Shandong Sports University, Jinan, China; 3 School of Sports Economics and Management, Hubei University of Economics, Wuhan, China; 4 Department of Physical Education, Wuhan Sports University, Wuhan, China; 5 Department of Basic Science, Wuchang Shouyi University, Wuhan, China; 6 Hubei Sports and Health Innovation and Development Research Center, Wuhan, China

Correction to: *Scientific Reports* 10.1038/s41598-024-57719-2, published online 25 March 2024

The original version of this Article contained an error in Affiliation 1, which was incorrectly given as ‘Department of Sports Big-Data Research Center, Wuhan Sports University, Wuhan, China’. The correct affiliation is listed below:

Sports Big-data Research Center, Wuhan Sports University, Wuhan, China.

Additionally, an affiliation was omitted for Yaping Zhong. Their correct affiliations are listed below.

Sports Big-data Research Center, Wuhan Sports University, Wuhan, China.

Hubei Sports and Health Innovation and Development Research Center, Wuhan, China.

The original Article has been corrected.

